# Therapeutic targeting of HCMV-encoded chemokine receptor US28: Progress and challenges

**DOI:** 10.3389/fimmu.2023.1135280

**Published:** 2023-02-13

**Authors:** Christian Berg, Mette M. Rosenkilde

**Affiliations:** Laboratory for Molecular Pharmacology, Department of Biomedical Sciences, Faculty of Health and Medical Sciences, University of Copenhagen, Copenhagen, Denmark

**Keywords:** HCMV (human cytomegalovirus), US28, targeting, drug development, small molecule, single-domain antibodies (sdAb), fusion toxin protein, viral chemokine receptor

## Abstract

The pervasive human cytomegalovirus (HCMV) causes significant morbidity in immunocompromised individuals. Treatment using the current standard-of-care (SOC) is limited by severe toxic adverse effects and anti-viral resistance development. Furthermore, they only affect HCMV in its lytic phase, meaning viral disease is not preventable as latent infection cannot be treated and the viral reservoirs persist. The viral chemokine receptor (vCKR) US28 encoded by HCMV has received much attention in recent years. This broad-spectrum receptor has proven to be a desirable target for development of novel therapeutics through exploitation of its ability to internalize and its role in maintaining latency. Importantly, it is expressed on the surface of infected cells during both lytic and latent infection. US28-targeting small molecules, single-domain antibodies, and fusion toxin proteins have been developed for different treatment strategies, e.g. forcing reactivation of latent virus or using internalization of US28 as a toxin shuttle to kill infected cells. These strategies show promise for providing ways to eliminate latent viral reservoirs and prevent HCMV disease in vulnerable patients. Here, we discuss the progress and challenges of targeting US28 to treat HCMV infection and its associated diseases.

## 1 HCMV disease and vCKR US28

The G protein-coupled receptor (GPCR) family comprises a vast number of receptors, which are involved in diverse aspects of cell signaling in the body. Some viruses encode homologs of GPCRs, including viral chemokine receptors (vCKRs), with distinct roles in infection, cardiovascular disease, and various types of cancers (1). The herpesvirus family is particularly adept at chemokine mimicry with several members carrying and maintaining viral chemokines, receptors, and chemokine-binding proteins (2). Human cytomegalovirus (HCMV, or HHV-5) is a pervasive herpesvirus that infects more than half the population on a global scale (3). Infection is typically transmitted during early childhood and leads to life-long latency from where HCMV sporadically reactivates throughout its host’s lifetime, thus maintaining and further transmitting the infection (4). While HCMV infection is largely subclinical in immune-competent individuals, both primary infection and reactivation of latent virus reservoirs cause significant morbidity and mortality in immunocompromised individuals (5). Because of its omnipresence and clinical significance in vulnerable patient groups, the adverse impact of HCMV disease is substantial. Congenital CMV disease, affecting 0.5-1% of live births and causing a wide range of developmental disorders (6, 7), including sensorineural hearing loss, vision impairment, and intellectual disability, has been ranked as one of the highest priority target diseases for vaccine development (8). In transplant recipients, HCMV is the most common and impactful viral infection causing debilitating and difficult-to-manage disease post-transplantation, increasing the risk of graft rejection and mortality (9). Furthermore, HCMV has been linked to cancers, the most well-supported being glioblastomas (GBM) (10–12), and cardiovascular disease (13, 14). The current standard-of-care (SOC) treatment (**Table 1**) consists of DNA synthesis inhibitors such as ganciclovir and foscarnet (15), but their use is limited by significant toxic adverse effects and can be impaired by viral resistance development when used in long-term regimens (16). Furthermore, the HCMV-specific terminase inhibitor letermovir was recently approved for prophylaxis in recipients of allogeneic stem cell transplants (17). Treatment of HCMV infection is further challenged by these replication inhibitors not affecting the virus in its latent stage where viral transcriptional activity is silenced, and replication is halted (18, 19). This implies that reactivation is not preventable as only lytic infection is treatable and the latent virus reservoirs persist (20).

**Table 1:** Overview of current standard-of-care drugs and the novel US28-targeting strategies under development, including modes of action (MOA), treatment effects and therapeutic applications.

	Drug/ Modality	MOA	Effect	Approved for^1^/ Therapeutic potential	Infection stage
**Current therapeutics**	Ganciclovir/ valganciclovir	Viral DNA polymerase inhibitor, activated by HCMV protein kinase UL97^2^	Replication inhibition	Prophylaxis and treatment of HCMV diseases in immunocompromised adults	Lytic
	Foscarnet	Viral DNA polymerase inhibitor	Replication inhibition	HCMV retinitis in people living with HIV/AIDS^3^	Lytic
	Cidofovir	Viral DNA polymerase inhibitor	Replication inhibition	HCMV retinitis in people living with HIV/AIDS^3^	Lytic
	Letermovir	HCMV terminase complex inhibitor (encoded by HCMV genes UL56, UL51 and UL89)	Replication inhibition	HCMV prophylaxis following allogeneic hematopoietic stem cell transplantation (allo-HSCT)	Lytic
	Maribavir	HCMV protein kinase UL97 inhibitor^2^	Replication inhibition	Treatment refractory post-transplant HCMV disease	Lytic
**Novel US28-targeting strategies**	Small molecules	Inhibition of vGPCR US28 constitutive signaling	Viral reactivation ➔ exposure to targeted killing by the immune system	Reducing latent HCMV load prior to immunosuppression (e.g. cancer patients and transplant recipients) Anti-proliferative treatment of US28+ GBM tumors	Latent
	Single-domain antibodies^4^	Inhibition of vGPCR US28 constitutive signaling	Viral reactivation ➔ exposure to targeted killing by the immune system	Reducing latent HCMV load prior to immunosuppression (e.g. cancer patients and transplant recipients) Anti-proliferative treatment of US28+ GBM tumors	Latent
		Photosensitizer-conjugate	Targeted killing of infected cells	*Ex vivo* clearance of HCMV infection in donor organs Treatment of US28+ cancers	Lytic and latent
	Fusion toxin proteins	Molecular trojan horse for intracellular toxin delivery through vGPCR US28	Targeted killing of infected cells	*Ex vivo* clearance of HCMV infection in donor organs Treatment of US28+ cancers	Lytic and latent

^1^FDA approved applications of the drugs.

^2^Combination of ganciclovir and maribavir is contraindicated as ganciclovir requires activation by UL97 that maribavir inhibits.

^3^Traditionally also used off-label as second-line treatment for ganciclovir-resistant HCMV infection.

^4^Also envisioned fused to a toxin moiety as an FTP (US patent US 2022/0324947 A1 (2022) 2020/08/05).

HCMV carries a large genome of ~235 kb linear double-stranded DNA comprising more than 750 translated open reading frames (ORFs) (21). Although most have unknown functions, more than 40 interact with the immune system (22, 23). In this vast genetic landscape, several genes with homology to components of the chemokine system has been identified (2). These include viral chemokines (UL146 and UL147), chemokine-like envelope proteins (UL128 and UL130), secreted chemokine binding proteins (UL21.5), and chemokine receptor homologs (US27, US28, UL33, and UL78). The best studied of these is the vCKR US28. This broad-spectrum receptor is expressed on the surface of HCMV infected cells, both in the lytic and latent phase (24, 25), and was initially recognized as a chemokine scavenging protein due to its promiscuous binding of many endogenous chemokines (26). Chemokine binding results in fast internalization of the ligand-receptor complex in a dynamin-dependent but arrestin-independent manner (27, 28), the internalized chemokine undergoes lysosomal degradation, and US28 is recycled to the cell surface where the process can repeat (24). This cycle theoretically removes pro-inflammatory chemokines from the extracellular environment at infection sites and promotes viral immune evasion, however, its biological significance has not been clearly established. It has been suggested that this effect is more pronounced during latency as the overabundance of extracellular chemokines in the lytic phase exceeds the scavenging capacity of US28 expressing cells (29). A well-established role of US28 during the latent phase is maintaining latency by subduing expression of the major immediate early promotor (MIEP). This effect is in part mediated by suppression of the mitogen-activated protein kinase (MAPK) and nuclear factor kappa-light-chain-enhancer of activated B cells (NF-κB) signaling pathways (25). Attenuation of MAPK signaling was recently shown to be the result of US28 interacting with the ephrin receptor A2 (EphA2) (30), whereas NF-κB signaling is subdued through rapid downregulation of interferon gamma inducible protein 16 (IFI16) by US28 (31). These functions underline an importance for HCMV immune evasion.

From a structural point of view, US28 overall resembles other class A GPCRs (1, 32–34). Of note, recent years’ advancements in structural biology of membrane proteins using for instance cryo-EM, have resulted in a multitude of structures across class A (and class B1) GPCRs. Often more than one structure for each receptor is defined, thereby capturing the receptors in various conformational states (35, 36). For US28, an apo-structure as well as complexes with CX_3_CL1 and a G protein-biased CX_3_CL1 variant have been solved (1, 32–34). Together, these have shed light on helical connectivity and the role of various receptor domains and microswitches for US28 activity. Overall, the structural alterations result in a differentiation of US28 from its homologous endogenous chemokine receptors (CX_3_CR1 and CCR1, CCR2, and CCR5) in terms of i) a broader chemokine recognition pattern (26, 37–39); ii) a broader activation profile, not only including Gαi like the endogenous receptors, but also other G proteins such as Gαq (40, 41); iii) a fast and constitutive internalization (24, 27, 28, 42, 43); and iv) a robust ligand-independent signaling (1, 44).

**Figure 1:**
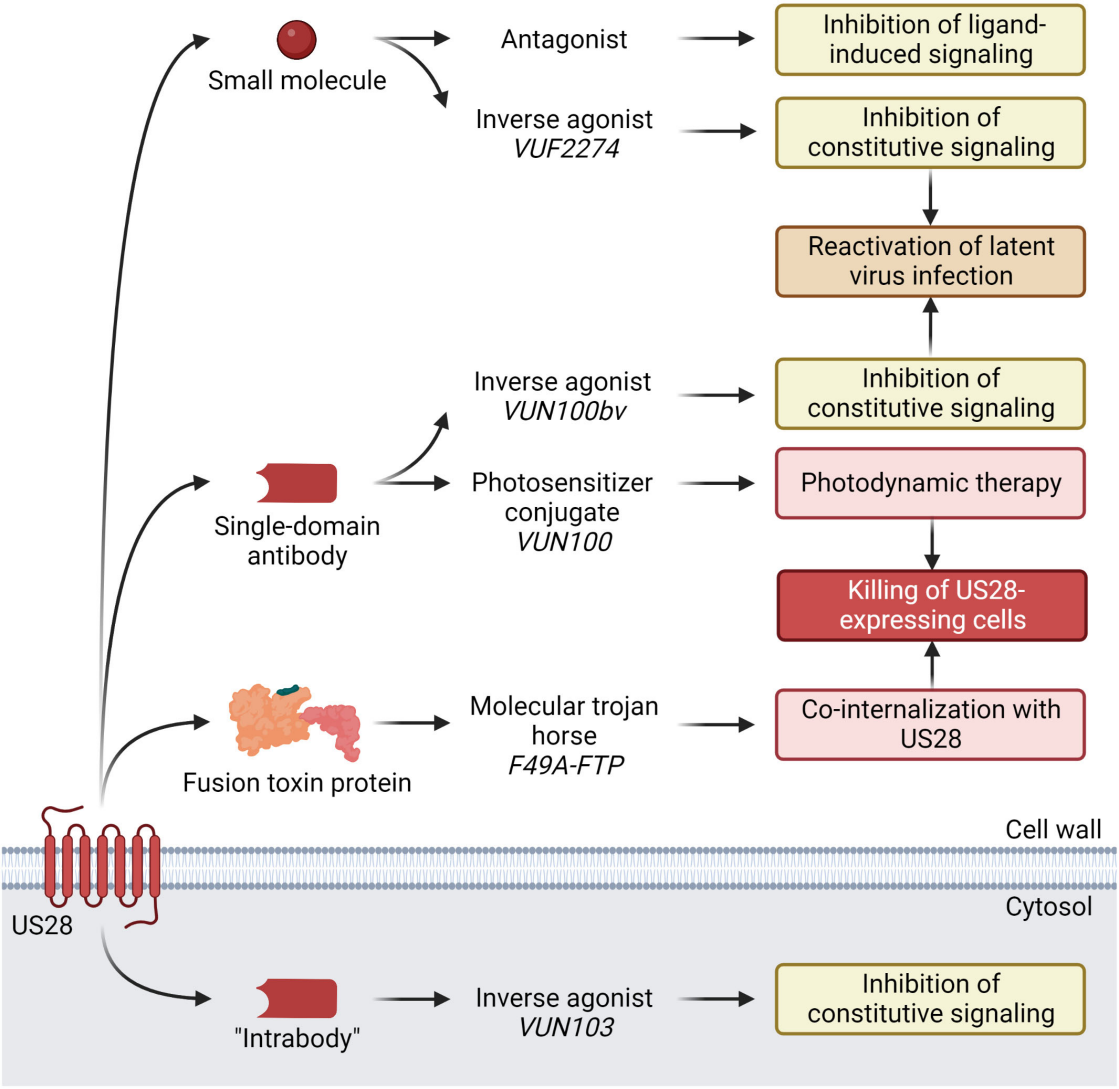
Current anti-HCMV US28-targeting modalities under development, discovered compounds, and their demonstrated effects. Small molecule VUF2274 and sdAb VUN100bv act as inverse agonists, i.e. inhibitors of US28 constitutive signaling. Attenuation of US28 signaling results in activation of the major immediate early promotor (MIEP), which leads to HCMV reactivation from latency. Another sdAb, VUN100, in conjugation with a photosensitizer binds to US28 on the surface of HCMV-infected cells. Upon stimulation with near-infrared light, the sdAb-photosensitizer conjugate is activated, producing reactive oxygen species that cause cell death. F49A-FTP consists of a US28-specific chemokine domain and a Pseudomonas exotoxin A (PE) domain that are fused. Upon binding to US28, it is co-internalized with the receptor. Inside the HCMV infected US28-expressing cell, the PE domain is released by furin cleavage. PE inactivates the eukaryotic elongation factor-2 (eEF-2), which halts host cell protein synthesis, resulting in apoptosis and cell death. The “intrabody” VUN103 is a sdAb that targets an intracellular epitope of US28. By displacing Gαq, it inhibits the constitutive signaling of US28 and exerts anti-proliferative effects on US28+ GBM tumor growth. Created with BioRender.com.

In search of novel therapeutics targeting HCMV infection, a new approach has emerged in recent years where US28’s role as a surface protein during both lytic and latent infection is exploited through its ability to internalize and role in viral reactivation. The strategies for therapeutic targeting of US28 so far encompass three distinct modalities (**Figure 1** and **Table 1**): small molecules, single-domain antibodies (sdAbs, so-called nanobodies), and fusion toxin proteins (FTPs). The inherent strengths, challenges, and potential clinical indications of these approaches will be discussed in this review.

## 2 Progress on drug targeting of US28

### 2.1 Small molecules targeting US28

A growing number of GPCR structures have facilitated the discovery of interacting small molecule compounds. For US28, the first compounds were discovered based on homology comparison to endogenous CKRs with known small molecule ligands as these were presented before the first US28 structure was solved. Several small molecule ligands acting as neutral antagonists or inverse agonists have displayed promising results in attenuating US28 signaling at micromolar concentrations (45). Among these, VUF2274 demonstrated the highest potency on US28 acting as an inverse agonist and interfered with CCL5 binding (45). The compound was observed to induce reactivation of latent HCMV infection potentially exposing it to the immune system (25), however, VUF2274 was originally discovered as a CCR1 antagonist (46) implying a selectivity issue if used as a drug. In search of potential drug candidates with limited cross-reactivity to endogenous receptors, two subsequent studies surveyed small molecule libraries based on VUF2274 (47, 48). Out of the latest study, several compounds emerged with agonistic or inverse agonistic profiles in Gαq-mediated signaling and capable of displacing CCL2 and CCL4, such as compound 56, 64 and 67 (48). These molecules were suggested as scaffolds for further development, but no advances on small molecules targeting US28 have been made since. Together, these studies demonstrate that US28, like other class A GPCRs, is highly targetable by small molecules though their clinical relevance as anti-HCMV therapeutics remains to be determined.

### 2.2 Single-domain antibodies to modulate US28 signaling

Apart from using small molecules to manipulate US28 activity, sdAbs are currently under investigation (49–52). Initially, a sdAb with sub-micromolar affinity to US28 was refined to create a bivalent sdAb with sub-nanomolar affinity. This compound partially inhibited ligand-dependent and constitutive US28 activity, leading to a reduction in US28+ GBM cell growth *in vitro* and *in vivo* (49), which shows therapeutic potential as US28 constitutive signaling can drive GBM proliferation (49). Subsequently, a US28-specific sdAb, VUN100, with nanomolar affinity for use in photodynamic therapy after conjugation with a photosensitizer was designed. Besides showing improved CX_3_CL1 displacement compared to its predecessor, this compound displayed potent cytotoxicity *in vitro* on US28+ GBM cells (50). VUN100 was further refined into a bivalent version (VUN100bv) with improved affinity, acting as a partial inverse agonist inhibiting constitutive US28 signaling by 50% (51). This resulted in partial reactivation of HCMV in latently infected primary CD14+ monocytes, which lead to the hypothesis that VUN100bv could be used as a therapeutic in a “shock-and-kill” strategy where latent viral reservoirs are forced into lytic replication and subsequently killed by the host immune system. A fourth study described the generation of a sdAb (VUN103) targeting an intracellular epitope (“intrabody”) that through displacement of G proteins completely inhibits constitutive US28 signaling and attenuates spheroid growth of U251 glioblastoma cells (52). Together, this set of studies underlines the possibility of US28 targeting and modulation through sdAbs. In the clinic, a sdAb-based strategy could potentially be used to treat HCMV diseases through attenuation of US28 signaling, leading to partial viral reactivation. This could expose the latent infection and improve the immune system’s ability to combat the virus, potentially combined with existing anti-HCMV drugs.

### 2.3 US28-binding fusion toxin proteins kill infected cells

A different approach to novel therapeutics for HCMV disease has focused on the development of a US28-specific FTP (an immunotoxin strategy) (53–55). In this case, the drug is not intended to modulate US28 signaling but rather to kill US28-expressing cells. To generate a US28-targeting FTP, the preferentially US28-binding chemokine CX_3_CL1 was fused to a modified version of the *Pseudomonas* exotoxin A (PE) lacking the cell entry moiety (53). Taking advantage of US28’s constitutive internalization, the bound FTP is shuttled inside the cell where the toxin domain is released (53, 54). PE inactivates eukaryotic elongation factor-2 (eEF-2) by ADP-ribosylation which abolishes host cell protein synthesis, resulting in apoptosis and inevitable cell death (56). As CX_3_CL1 also binds the endogenous receptor CX_3_CR1, a mutated variant with high US28 selectivity (F49A-FTP) was generated. Exploiting the ubiquitous expression profile of US28 throughout both the lytic and latent cycle of HCMV infection, this FTP displayed potent and selective killing of infected cells in both stages (53, 55). The efficient elimination of HCMV-infected cells indicates a potential use in treatment of HCMV-associated diseases as demonstrated in patient-derived HCMV-infected CD34+ progenitor cells *in vitro*, forming the basis for a therapeutic strategy for eliminating latently infected cells before hematopoietic stem cell transplantation (55). Additionally, it showed efficacy on ganciclovir-resistant HCMV strains (53) thereby suggesting a use-case in clinical settings of treatment failure due to viral ganciclovir resistance. Following a successful trial-run (57), F49A-FTP was recently shown to reduce the load of latent HCMV by 80% in an *ex vivo* lung perfusion system (58), showcasing the potential for *ex vivo* elimination of HCMV in solid organ transplantations. These reports support a novel approach of eradicating latent virus reservoirs, which could prove particularly useful in organ transplantation settings provided improved clinical outcomes can be demonstrated.

## 3 Challenges of targeting US28 to treat HCMV diseases

### 3.1 Bridging the gap between bench and bedside

The reports on US28-targeting compounds are promising but crossing the gap between laboratory observations and *in human* effects is notoriously challenging for HCMV. The virus is highly adapted and species-specific after millions of years of co-evolution with its host (59), making HCMV significantly different from other species’ CMVs in its genetic content and immune modulation. The lack of a proper animal model for replicating *in vitro* effects *in vivo* is a persistent challenge in the field. Transgenic animal models have been applied with success, e.g. insertion of HCMV US28 into murine CMV (53, 60–62), however, findings from transferring HCMV-specific genes to another species’ CMV are difficult to translate to humans and should always be considered with caution. Even though the distance between bench and bedside is increased by the lack of animal models for HCMV-associated diseases, US28 has the favorable position of a surface protein with basal, exploitable functionalities combined with homologies to endogenous class A GPCRs, which are inherently good drug targets (63).

Still, for transplantation-associated HCMV diseases, perhaps a better option is to utilize latently infected human organs unfit for clinical use in *ex vivo* systems (58). Here, the bigger hurdle is detecting and quantifying the latent HCMV load. Albeit not yet fully understood, latency is known to be established in a small fraction of CD34+ hematopoietic progenitor cells (HPCs) and CD14+ monocytes (64, 65), which are not abundant in most *ex vivo* organ settings. Additionally, gene transcription is minimal during latency (64). Reactivation assays have been described (58) but are time consuming, require steps of target cell extraction, viral reactivation, amplification using standardized cell cultures, and immunohistochemical staining of viral components, yielding more of an indirect measurement of HCMV activity. Modern techniques, such as RNA-seq, have shown promise in detecting latency transcripts (66) and may provide another approach to studying latency and *ex vivo* treatment effects. However, since reactivation is an inefficient process, genome- and transcript-based methods likely include abortive infections that will not reactivate. Alas, our current methods for detecting and quantifying the latent HCMV load and reactivation, and therefore evaluation treatment outcomes, are not ideal.

### 3.2 US28 genetic diversity

HCMV has a surprisingly diverse genome for a DNA virus displaying a high degree of sequence variability across many different genes including major immune modulators (67). For example, the chemokine-encoding UL146 gene is subject to extensive inter-strain diversity (68) that leads to structural and functional changes of the chemokine (69, 70). The US28 gene in contrast is quite conserved, strengthening its position as a therapeutic target (67). However, various genotypes have been observed (71–73), notably some with marked differences in the N-terminal (extracellular) tail of the receptor, which is important for chemokine binding. Indeed, molecular modeling has predicted changes in binding affinities of several endogenous chemokines to US28 variants (73). Variations of extracellular loops (ECLs) and the C-terminal (intracellular) tail have also been observed and, albeit less extensive, are not to be overlooked for their potential to alter US28 signaling. While the mechanism and biological significance are unclear, one study reported an increase in anti-CMV antibodies of renal transplant recipients carrying R267K-US28 (73). Additionally, antibody levels were reduced in HIV infected individuals carrying D170N-US28 and were accompanied by an increased HIV viral load and a reduction in sIFN-α/βR levels 12 months post initiation of anti-retroviral therapy (73). Functional differences between these naturally occurring variants remain unknown, but future research efforts exploring shifts in chemokine and drug binding along with signaling properties of the US28 variants will provide more knowledge. Furthermore, it is unclear to what extent these variants occur on a global scale as US28 genotyping studies and GenBank sequence deposits are limited. This combines to some uncertainties that should be addressed when progressing with US28-targeting drugs, as changes in drug affinities for US28 variants and downstream signaling can lead to altered drug effects. Additionally, treatment might induce US28 resistance mutations. These are risks that require clinical US28 sequencing before and during treatment to ensure and monitor the expected drug effects, however, clinically standardized tools to amplify and sequence US28 during latency is currently not available. Thus, these unknowns require attention when transitioning from lab to clinic.

### 3.3 What does it take to improve the clinical outcome?

The US28-targeting strategies discussed here rely on two distinct modes of action (MOA) (**Figure 1** and **Table 1**). For small molecules and sdAbs, a “shock-and-kill” strategy has been proposed where HCMV infection is forced from latent to lytic phase. This is achieved by inhibition of constitutive US28 signaling with an inverse agonist that leads to activation of MIEP which initiates viral reactivation (74). Once reactivation is induced, this strategy relies on the host immune system or a combination treatment with a replication inhibitor to clear the infection (25). This will in theory allow clearance of HCMV infected cells before dissemination of infection. While elegant in its conception, this strategy rises some safety concerns if used in immunocompromised patients. Forcing HCMV reactivation requires a degree of control over the infection that currently is not always possible as seen in patient groups where infection can flare up despite administration of SOC prophylaxis (15), such as transplant recipients. This potentially limits the usefulness of the “shock-and-kill” strategy to patients with somewhat competent immune systems, which could be envisioned in an early treatment for US28+ GBM tumors, or to reduce the latent HCMV load in other cancer patients and R+ transplant recipients prior to immunosuppressive therapy.

The MOA of sdAb-photosensitizer conjugates and FTPs is cell toxicity, which does not rely on a competent immune system or a combination treatment to finish the job. However, this advantage requires a highly US28-specific ligand domain to limit adverse toxic effects emerging from off-target binding. Indeed, the promiscuous binding of US28 (26) might make it an easier target but also increase the risk of non-specific compound effects from off-target receptors. Promisingly, F49A-FTP did not induce acute lung injury or changes in cytokine levels in an *ex vivo* lung perfusion setting (58). As for other immunotoxins, potential compartmentalization of the FTP combined with release of the toxin moiety will need to be addressed to ensure its safety. While the sdAb-photosensitizer conjugate has the benefit of requiring site-directed light activation, peripheral effects of reactive oxygen species resulting from photodynamic therapy could affect its viability in some cancer and transplantation settings. Lastly, clearing latent infection from donor organs prior to transplantation (55, 58) raises the question to what extent the viral reservoirs need to be reduced to influence the post-transplantation outcome. While early studies show that there is an association between the latent viral load and risk of recurrent infection (75, 76), it is unclear how the size of a latent HCMV reservoir in a donor correlates with the risk of reactivation and disease in the recipient, but it is not unthinkable that a small persistent HCMV pool can flare up to clinical significance in vulnerable patients. Overcoming these potential clinical challenges will be key to progression.

## 4 Concluding remarks

HCMV encodes three other genes with GPCR homology, US27, UL33, and UL78. These viral receptors are far less studied, and their functions and interaction partners remain largely unknown. UL33 and UL78 have been detected in some latency models *in vitro* whereas US27 is not expressed during latency (66) but has a role in viral dissemination in the lytic phase (77). Thus, US28 remains the prime target for novel therapeutics of HCMV-associated diseases.

Therapeutic targeting of CKRs has been a goal for more than 25 years. Despite the GPCR family being considered highly druggable, with nearly 500 successful drugs amounting to 34% of all FDA-approved drugs (63), strategies for developing treatments targeting CKRs have resulted in only three drugs approved for clinical use (Maraviroc, Plerixafor, and Mogamulizumab). Many more candidates have been tested and failed which shows that CKRs are not so straightforward targets as we initially had hoped. The reason is complex but can roughly be summed up to this—the chemokine system is highly promiscuous and redundant, vitally important for numerous biological processes, and disrupting it causes problems. Supporting this, the three approved drugs targeting CKRs are not designed to broadly alter inflammatory processes, but instead inhibits HIV-1 cell entry *via* CCR5 (78), promotes stem cell recruitment from the bone marrow *via* CXCR4 (79–81), and affects recruitment of a selected cell subset (regulatory T cells) to tumors *via* CCR4 (82).

On the quest for new CKR-targeting drugs, looking towards vCKRs could provide a solution for virus-associated diseases, but vCKR drug development is still in its youth. In this review, we have provided an update on HCMV US28 drug targeting and have discussed the major hurdles we currently face. Ongoing studies will reveal the further potential of the different US28-targeting strategies when progressing towards clinical adaptation.

## Author contributions

The authors have contributed equally to this review. All authors contributed to the article and approved the submitted version.
